# Defying gravity: Breath, beat and brain

**DOI:** 10.1113/EP093309

**Published:** 2025-10-12

**Authors:** Shigehiko Ogoh, Damian Miles Bailey

**Affiliations:** ^1^ Department of Biomedical Engineering Toyo University Asaka Japan; ^2^ Neurovascular Research Laboratory, Faculty of Life Sciences and Education University of South Wales Glamorgan UK

**Keywords:** arterial baroreflex, blood pressure, cerebral blood flow, orthostatic stress, respiration

1

Orthostatic tolerance reflects the capacity of the body to withstand gravitational stress, without dizziness, presyncope or syncope, when moving from a supine to an upright posture. It ensures adequate cerebral blood flow (CBF) and blood pressure (BP) stability despite venous pooling in the lower extremities and relies on rapid and integrated cardiovascular and respiratory reflexes, including the arterial baroreceptor reflex, the respiratory and skeletal muscle pumps and autonomic adjustments in heart rate, vascular tone and ventilation. Collectively, these mechanisms preserve cerebral bioenergetic function to prevent fainting and enable humans to sustain upright posture and mobility, a crucial feature of daily life in a bipedal species.

Upon standing, gravity causes 500–800 mL of blood to pool in the lower body, reducing left ventricular end‐diastolic filling, stroke volume (SV) and cardiac output by ≤20% (Harms et al., 1999). Orthostatic tolerance varies considerably between individuals, with reports indicating that endurance‐trained athletes experience greater reductions in SV (Levine, 1993) and attenuated carotid–cardiopulmonary baroreflex interactions during orthostatic challenges, which might contribute to orthostatic intolerance (Ogoh et al., 2003). This is linked to cardiac remodelling, increased central blood volume (CBV) and enhanced cardiac compliance, resulting in a steeper Frank–Starling curve. In patients diagnosed with vasovagal syncope, baroreflex failure is evident despite initially normal tilt responses. Jardine et al. (2025) demonstrated that these patients gradually exhibited a decline in baroreflex gain, leading to vasodilatation and presyncope, highlighting baroreflex function as a key determinant of orthostatic tolerance. Nevertheless, the mechanisms underlying inter‐individual differences remain incompletely understood. Respiratory function also contributes to orthostatic tolerance. Controlled slow breathing improves tolerance (Lucas et al., 2013); early animal studies demonstrated that carotid baroreceptor activation directly modulates tidal volume and ventilatory patterns (Brunner et al., 1982). In dogs with vagotomy and carotid body denervation, baroreceptor loading increased tidal volume while decreasing respiratory frequency and minute ventilation, whereas unloading had the opposite effect. These responses persisted even when mean arterial pressure was clamped at 100 mmHg, indicating a direct baroreceptor influence on respiration (Brunner et al., 1982). Consistently, Ogoh and colleagues (Ogoh, 2019; Ogoh et al., 2009, 2008) reported functional interactions between respiratory control and CBF regulation. Collectively, these findings suggest that respiratory control contributes to BP and CBF regulation during orthostatic stress and might underlie individual differences in tolerance.

The present investigation by Feeley et al. (2025) sought to extend previous findings on longer‐term, steady‐state responses. The study focused on the temporal dynamics of integrated cardiorespiratory and cerebrovascular responses during the first 2 min of acute lower‐body negative pressure (LBNP)‐induced CBV reduction. This approach offers unique insight, because early responses are most strongly associated with dizziness and adverse outcomes, compensatory mechanisms peak in this phase, and understanding rapid adjustments might help to guide clinical interventions (Juraschek et al., 2017). The study by Feeley et al. (2025) extends the existing body of knowledge with several important findings.

First, the respiratory response to LBNP appears largely independent of baroreflex activation. Unlike the animal experiments by Brunner et al. (1982), which showed that unloading of arterial baroreceptors decreased tidal volume and increased respiratory frequency, Feeley et al. (2025) observed no significant change in group‐level tidal volume during LBNP‐induced unloading of arterial baroreceptors. However, in the first 0–30 s, individuals with larger BP reductions tended to increase tidal volume, reflecting inter‐individual variability. These findings suggest that LBNP‐induced respiratory changes might occur independently of baroreflex interactions, although further mechanistic studies are required. These differences from animal data are likely to reflect species‐ and condition‐specific factors, such as intact vagal and chemoreceptor inputs, thoracic mechanics and cerebral influences. Additionally, reductions in regional CBF, particularly in the brainstem, might also contribute (Chapman et al., 1979).

A second key finding relates to the early onset phase (0–30 s) of orthostatic stress. During this period, as CBV decreased, tidal volume increased and was correlated with smaller reductions in arterial BP. A decrease in respiratory rate, coupled with increased tidal volume, enhances the respiratory pump, helping to preserve CBV and attenuate the fall in arterial BP. The negative intrathoracic pressure generated by slow respiration improves venous return to the right atrium, elevating SV (Brecher & Hubay, 1955; Innes et al., 1993).

An additional important observation concerns CBF regulation. At the onset of orthostatic stress, unloading of cardiopulmonary baroreceptors is known to increase sympathetic nerve activity, which redistributes CBV to help maintain CBF (Ogoh et al., 2015). Interestingly, Feeley et al. (2025) found that mild orthostatic stress failed to elevate CBF, implying that respiratory adjustments, probably combined with cerebral autoregulation, were sufficient to maintain bioenergetic function. Under more severe stress, respiratory compensation might be inadequate, leading to reductions in arterial BP and CBF, ultimately predisposing to syncope.

Collectively, by examining the onset response, the study by Feeley et al. (2025) provides new insights into the complex, integrated mechanisms underlying orthostatic tolerance; mechanisms that steady‐state measurements alone have consistently failed to reveal. This important study not only advances our understanding of cardiorespiratory and cerebrovascular integration during orthostatic stress but also underscores the need for further research to elucidate these intricate mechanisms fully.

## AUTHOR CONTRIBUTIONS

Writing the first draft of the manuscript and revisions thereof: Shigehiko Ogoh and Damian Miles Bailey. Approving the final version submitted for publication and agreeing to be accountable for all aspects of the work in ensuring that questions related to the accuracy or integrity of any part of the work are appropriately investigated and resolved: Shigehiko Ogoh and Damian Miles Bailey. Both persons designated as authors qualify for authorship, and all those who qualify for authorship are listed.

## CONFLICT OF INTEREST

D.M.B. is Editor‐in‐Chief of *Experimental Physiology* and outgoing Chair of the Life Sciences Working Group and outgoing member of the Human Spaceflight and Exploration Science Advisory Committee to ESA. D.M.B. is a current member of the ESA‐HRE‐Biology Panel and Space Exploration Advisory Committees to the UK and Swedish National Space Agencies. S.O. is a Senior Editor of *Experimental Physiology*.
